# Are degenerative findings detected on traction MR arthrography of the hip associated with failure of arthroscopic femoroacetabular impingement surgery?

**DOI:** 10.1007/s00330-023-10419-3

**Published:** 2023-11-20

**Authors:** Till D. Lerch, Andreas K. Nanavati, Alexander F. Heimann, Malin K. Meier, Simon D. Steppacher, Moritz Wagner, Alexander Brunner, Peter Vavron, Ehrenfried Schmaranzer, Joseph M. Schwab, Moritz Tannast, Florian Schmaranzer

**Affiliations:** 1 Department of Diagnostic-, Interventional- and Pediatric Radiology, Inselspital, Bern University Hospital, University of Bern, Freiburgstrasse, 3010 Bern, Switzerland; 2 Department of Orthopaedic Surgery and Traumatology, Inselspital, Bern University Hospital, University of Bern, Freiburgstrasse, 3010 Bern, Switzerland; 3 Department of Orthopaedic Surgery and Traumatology, HFR – Cantonal Hospital, University of Fribourg, Chemin Des Pensionnats 2-6, 1700 Fribourg, Switzerland; 4 Department of Orthopaedic Surgery and Traumatology, District Hospital St. Johann in Tirol, Bahnhofstrasse 14, 6380 St. Johann in Tirol, Austria; 5 Department of Radiology, District Hospital St. Johann in Tirol, Bahnhofstrasse 14, 6380 St. Johann in Tirol, Austria; 6 Department of Radiology, Balgrist University Hospital, Faculty of Medicine, University of Zurich, Forchstrasse 340, 8008 Zurich, Switzerland

**Keywords:** MRI, Hip, MR arthrography, Hip arthroscopy, Labrum

## Abstract

**Objectives:**

To identify preoperative degenerative features on traction MR arthrography associated with failure after arthroscopic femoroacetabular impingement (FAI) surgery.

**Methods:**

Retrospective study including 102 patients (107 hips) undergoing traction magnetic resonance arthrography (MRA) of the hip at 1.5 T and subsequent hip arthroscopic FAI surgery performed (01/2016 to 02/2020) with complete follow-up. Clinical outcomes were assessed using the International Hip Outcome Tool (iHOT-12) score. Clinical endpoint for failure was defined as an iHOT-12 of < 60 points or conversion to total hip arthroplasty. MR images were assessed by two radiologists for presence of 9 degenerative lesions including osseous, chondrolabral/ligamentum teres lesions. Uni- and multivariate Cox regression analysis was performed to assess the association between MRI findings and failure of FAI surgery.

**Results:**

Of the 107 hips, 27 hips (25%) met at least one endpoint at a mean 3.7 ± 0.9 years follow-up. Osteophytic changes of femur or acetabulum (hazard ratio [HR] 2.5–5.0), acetabular cysts (HR 3.4) and extensive cartilage (HR 5.1) and labral damage (HR 5.5) > 2 h on the clockface were univariate risk factors (all *p* < 0.05) for failure. Three risk factors for failure were identified in multivariate analysis: Acetabular cartilage damage > 2 h on the clockface (HR 3.2, *p* = 0.01), central femoral osteophyte (HR 3.1, *p* = 0.02), and femoral cartilage damage with ligamentum teres damage (HR 3.0, *p* = 0.04).

**Conclusion:**

Joint damage detected by preoperative traction MRA is associated with failure 4 years following arthroscopic FAI surgery and yields promise in preoperative risk stratification.

**Clinical relevance statement:**

Evaluation of negative predictors on preoperative traction MR arthrography holds the potential to improve risk stratification based on the already present joint degeneration ahead of FAI surgery.

**Key Points:**

*• Osteophytes, acetabular cysts, and extensive chondrolabral damage are risk factors for failure of FAI surgery.*

*• Extensive acetabular cartilage damage, central femoral osteophytes, and combined femoral cartilage and ligamentum teres damage represent independent negative predictors.*

*• Survival rates following hip arthroscopy progressively decrease with increasing prevalence of these three degenerative findings.*

**Graphical Abstract:**

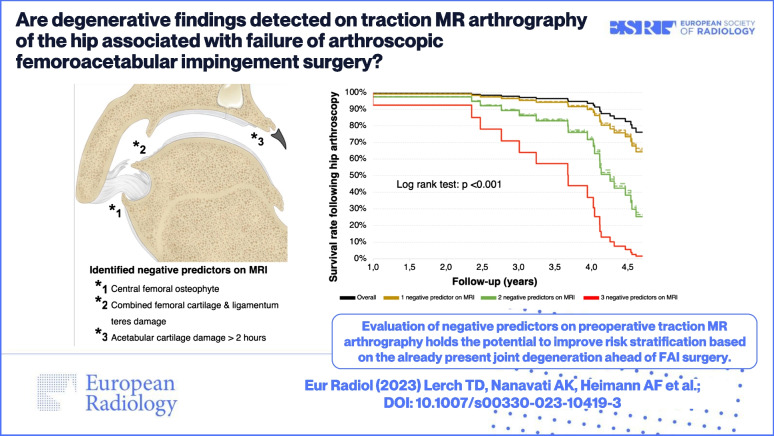

## Introduction

In femoroacetabular impingement (FAI), the acetabular rim and proximal femur are in abnormal contact with each other, leading to hip pain, restricted movement, chondrolabral damage, and risk of total hip replacement at an early age [1–3]. While pelvis radiographs are important to assess the shape and orientation of the acetabulum, magnetic resonance imaging (MRI) has emerged as the modality of choice to assess chondrolabral damage [2, 4]. Despite the overall success of FAI surgery, a good outcome with durable preservation of the native hip joint cannot be achieved in up to 18% of patients within one year [5]. In fact, a successful outcome of FAI surgery usually hinges on the degree of already present intra-articular damage aside from advanced patient age [5, 6]. Preoperative MRI is performed to identify patients who are poor candidates for surgery. Yet, despite being commonly recommended in the preoperative workup ahead of FAI surgery, the prognostic value of hip MRI remains poorly understood [7]. Few studies have evaluated preoperative imaging risk factors based on non-contrast MRI [8, 9] or direct MR arthrography (MRA) [10] of the hip, showing the potential of degenerative MRI findings to improve patient selection. However, assessment of chondral damage on non-contrast MRI and MRA is challenging due to the tight coaptation of the femoral head and acetabulum [11]. MRA with axial leg traction has been introduced to allow for better central compartment visualization and improved detection of chondrolabral lesions [11, 12]. However, its prognostic value to predict the outcome of FAI surgery is currently unknown.

Thus, we aimed to assess the association of degenerative MRI findings detected on traction MRA of the hip for predicting failure of arthroscopic FAI surgery.

## Material and methods

### Patients

**Fig. 1:**
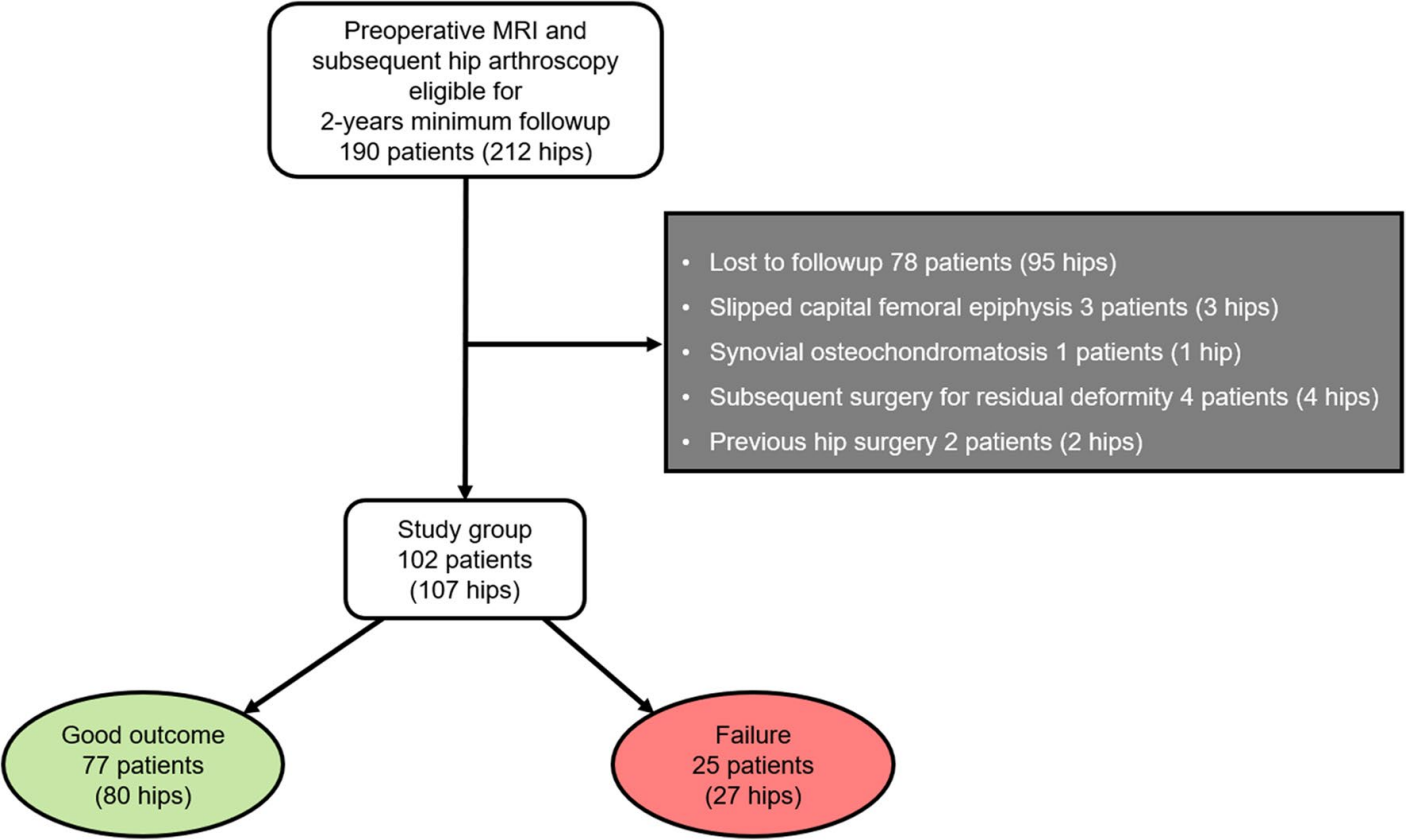
Flow chart with inclusion and exclusion criteria. Clinical endpoint for allocation to the “failure” group was defined as subsequent total hip arthroplasty or patients not achieving the acceptable symptom state of the international Hip Outcome tool-12 questionnaire of 60 points

Following institutional review-board approval under a waiver for written informed consent, a retrospective cohort study was performed of patients undergoing preoperative radiographs, traction MRA, and subsequent arthroscopic FAI correction. The study was performed at a primary hospital in Austria with a referral center for hip arthroscopy between 01/2016 and 02/2020. Patients were referred to MRI following a clinical diagnosis of FAI, which was established by one arthroscopic hip surgeon based on a positive impingement test, a > 3-month history of symptoms, and radiographic findings suggestive for FAI [4, 7]. Review of the institutional surgical database yielded 190 patients (212 hips) undergoing arthroscopic FAI surgery and preoperative traction MRA between 01/2016 and 02/2020. Patients were excluded if they did not complete patient-reported outcome scores at follow-up and had a history of slipped capital femoral epiphysis (SCFE), synovial osteochondromatosis, previous hip surgery, or revision hip surgery for residual osseous deformity. Following exclusion, 102 patients (107 hips) composed the final study cohort (Fig. 1).

### Diagnostic imaging

Preoperative anteroposterior pelvis- and Dunn 45° views were obtained in a supine position. Preoperative MRA using a large, flexible body coil was performed at 1.5 T (Aera; Siemens Healthcare), with intra-articular injection of either 15–20 mL of diluted MR contrast agent before January 2018 (gadopentetate dimeglumine, 2.0 mmol/L; Magnevist; Bayer Healthcare) or 15–20 mL of 0.9% NaCl solution from January 2018 on (Fresenius Kabi, Austria). Injection was performed under fluoroscopic guidance using 1–2 mL iodinated contrast agent (iopamidol, 200 mg/mL; Iopamiro 200; Bracco) and an additional injection of 2–5 mL local anesthetic (ropivacaine hydrochloride; 2 mg/mL; Ropinaest; Gebro Pharma). Leg traction during MRI was applied according to our institutional protocol utilizing a previously described approach and traction apparatus (TRACView; Menges Medical) [11]. A supporting plate for stabilization of the contralateral leg, a weight (adjusted to patients’ constitution: 15 kg for patients < 60 kg, 18 kg for patients 60–80 kg, 23 kg for patients > 80 kg) attached to a cable pulley connected to the ankle brace were used [11]. The MRI protocol consisted of proton density-weighted turbo-spin-echo sequences without fat saturation acquired under leg traction in sagittal-, coronal-, axial-oblique-, and radial orientation: repetition time/ echo time of 2460/ 13 ms, matrix of 512 × 512, field of view of 180 mm, flip angle of 150°, slice thickness of 3 mm and bandwidth of 130 Hz/Px. Overall imaging time under leg traction was 14–16 min. In addition, fast T1-w DIXON based sequences of the pelvis and distal femoral condyles were acquired without leg traction for measurement of femoral torsion: repetition time/ echo times of 6.7/ 2.4 and 4.8 ms, matrix of 320 × 320, field of view of 380 mm, flip angle of 10°, slice thickness of 3 mm and bandwidth of 470 Hz/Px.

### Arthroscopic hip surgery and clinical follow-up

Arthroscopic FAI surgery was performed by one orthopedic surgeon with 13 years of experience and included femoral- and acetabular osteochondroplasty in most patients (Table 1). To assess clinical outcome, patients were contacted electronically and asked to complete a survey. This included the short version of the International Hip Outcome Tool (iHOT-12) [13] and whether subsequent surgery for osseous deformity correction or total hip arthroplasty (THA) had been performed. Clinical endpoint for failure of FAI surgery was defined as patients either undergoing subsequent THA during the follow-up period, or not achieving the patients’ acceptable symptom state of the iHOT-12 of 60 points. This threshold has been previously validated and corresponds to a nearly normal reported function and at least a 50% satisfaction rate according to clinical validation studies [14, 15]. The absence of any of these endpoints was equivalent to a good clinical outcome.

**Table 1:** Demography and radiography of the study population

Parameter	Mean ± SD/ hips (%)
Patients (hips)	102 (107)
Age (years)	33 ± 10
Female (%)	42 (40)
Follow-up time (years) Surgical procedure	3.7 ± 0.9
Cam resection Labral refixation Labrum debridement Subtrochanteric derotational osteotomy	92 (86) 63 (59) 44 (41) 2 (2)
Tönnis grade of osteoarthritis > 0	39 (36)
Alpha angle radial MRI (°)	71 ± 9
Neck shaft angle (°)	129 ± 5
Femoral torsion (°)	29 ± 10
Lateral center edge angle (°)	28 ± 6
Acetabular index (°)	7 ± 5
Cross over sign	57 (53)
Posterior wall sign	58 (54)
Ischial spine sign	27 (25)

*SD* standard deviation

### Image analysis

**Fig. 2:**
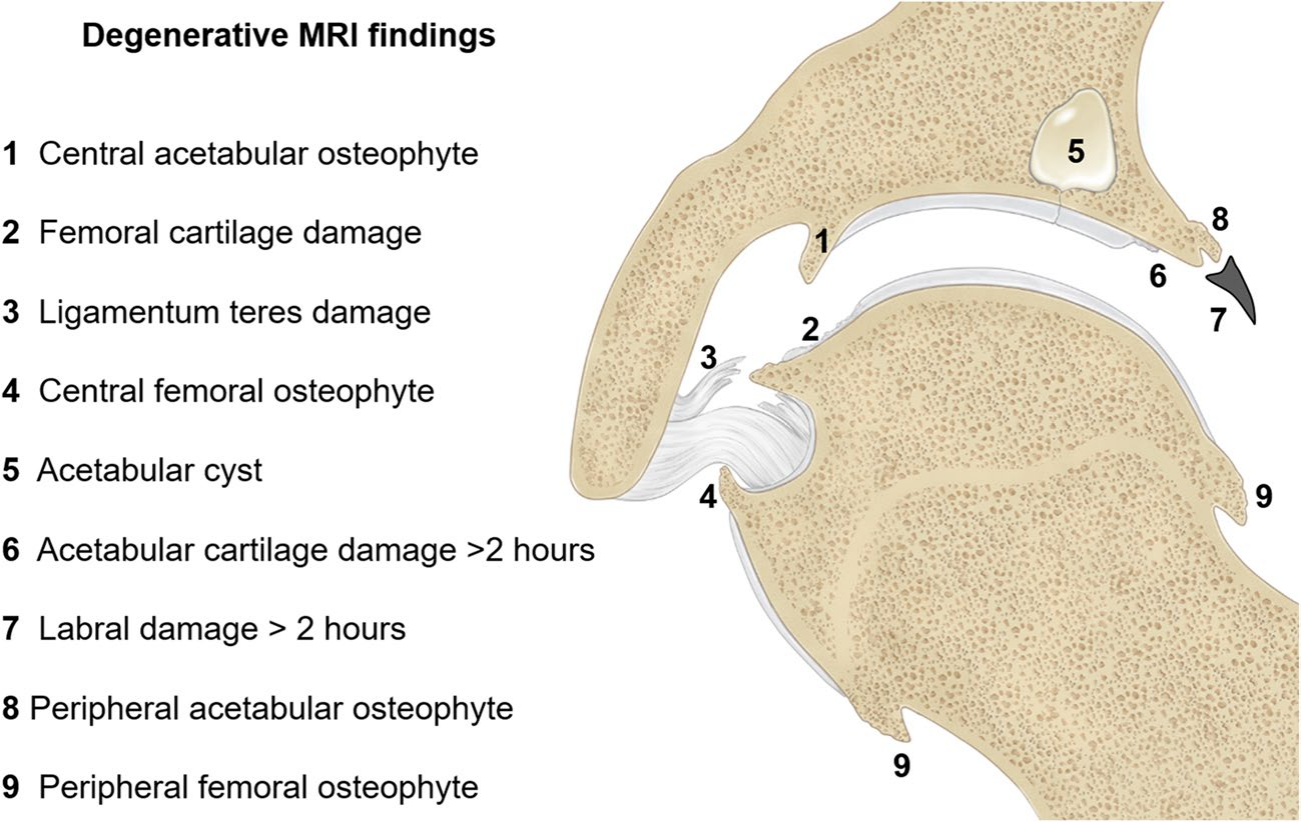
Schematic illustration showing the 9 degenerative findings evaluated on preoperative traction MR arthrography

A radiologist (E.S.) with 13 years of experience in hip imaging performed the readings blinded to the clinical outcome. Preoperative radiographs were examined for Tönnis grade of osteoarthritis, lateral center edge (LCE) angle, acetabular index, acetabular retroversion signs (ischial wall-, crossover-, posterior wall sign) [7, 16]. Alpha angle was measured on radial MRI [7]. Femoral torsion was measured on axial images of the pelvis and femoral condyles according to the Murphy method [17]. Joint damage on MRA was assessed with 9 parameters (Fig. 2): labrum damage > 2 h on the clockface (labral base tear, tear within labrum substance, complex labrum tear), femoral cartilage damage (delamination, thinning, defect), ligamentum teres damage (tear/ hypertrophy) [18], acetabular rim cyst, peripheral-/ central “perifoveolar” femoral osteophytes, and peripheral acetabular/ central “acetabular fossa” osteophytes [10, 19]. The presence of a central femoral osteophyte was defined as > 2 mm [19]. No size cutoff was used for the remaining osteophytes due to the lack of established criteria. The presence of acetabular cartilage damage (delamination, thinning, defect) > 2 h on the clock face was assessed due to its previously reported association with failure of FAI surgery [10, 20]. Femoral cartilage damage was not differentiated regarding extension < 2/ > 2 h on the clock face. MR image analysis was repeated by a second radiologist (F.S.) with 8 years of experience. For potential combinations of degenerative lesions (e.g., femoral cartilage associated with ligamentum teres damage), the same aforementioned criteria for lesion grading were used. For subsequent statistical analysis, the lesion grades were dichotomized into the presence/ absence of the corresponding lesion.

### Statistical analysis

Following testing for normal distribution with Kolmogorov–Smirnov tests, the paired t-tests were used for comparison of continuous data. The Fisher exact test was used for comparison of dichotomous variables. Single-factor, direct Cox regression analysis with calculation of the hazard ratios (HR) and 95% confidence intervals (CI) was applied to evaluate the association between joint damage parameters on MRI and failure after FAI surgery, as well as between the known negative predictors age > 40 years and Tönnis grade > 0 and failure after FAI surgery. To identify negative MRI predictors, uni- and multivariate Cox-regression analysis with calculation of HR and 95% CI for the retained parameters was performed. Based on the retained parameters of the stepwise multivariate Cox regression analysis, a Kaplan–Meier survival curve was generated, and global difference between curves was assessed using the log-rank test.

Interrater reliability of degenerative MRI findings was assessed using Cohen’s kappa (*κ* values ≤ 0 = no-, 0.01–0.2 = slight-, 0.21–0.4 = fair-, 0.41–0.6 moderate-, 0.61–0.8 substantial-, 0.81–1 almost perfect agreement) [21]. *p* values < 0.05 were used to determine statistical significance. Statistical analysis was performed with GraphPad Prism (Version 9.1, GraphPad Software).

## Results

### Patient characteristics

Of the 190 patients (212 hips) with preoperative traction MRA and subsequent arthroscopy at our institution, 78 patients (95 hips) were lost to follow-up. Three patients (3 hips) were excluded due to a history of slipped capital femoral epiphysis, 1 patient (1 hip) due to osteochondromatosis, 4 patients (4 hips) undergoing revision hip arthroscopy, and 2 patients (2 hips) due to previous surgery (Fig. 1). This left 102 patients (107 hips, mean age of 33 ± 10 years, 42 females; 40%) with a mean follow-up time of 3.7 ± 0.9 years (Table 1).

Of the 107 hips, 80 hips (75%) met the patient acceptable symptom state and did not convert to THA (Table 2). Among the patients with a preserved hip and good outcome, mean iHOT was 86 ± 12 points (95% CI, 84 to 89 points) at 3.6 ± 0.9 years follow-up. Twenty-seven hips (25%) met at least one endpoint. Ten hips (37% of those that failed) underwent total hip arthroplasty (9% overall conversion rate). Seventeen hips (63%) did not meet the patient acceptable symptom score with an overall mean iHOT of 44 ± 11 points (38 to 50 points), which was lower (*p* < 0.001) than in patients with good outcome (86 ± 12 points, 84 to 89 points). The proportion of hips in which MRA was performed with gadolinium was comparable between the hips that failed and hips with good outcomes (48%, 34 to 56% vs. 45%, 29 to 67%; *p* = 0.70). Patients that failed were older (38 ± 9 years, 34 to 42 years vs. 32 ± 10 years, 30 to 32 years; *p* = 0.01) had a higher percentage of hips with Tönnis grade > 0 (59%, 40 to 78% vs. 29%, 19 to 39%; *p* = 0.01) and had lower mean femoral torsion (25 ± 11°, 21 to 30° vs. 30 ± 10°, 28 to 32°; *p* = 0.046) than patients with good outcomes (Table 2).

**Table 2:** Comparison of radiographic and demographic characteristics of the study groups

Parameter	Good outcome mean ± SD/ hips (%)	95% CI	Failure mean ± SD/ hips (%)	95% CI	*p* value
Number of hips	80 (75)	-	27 (25)	-	**-**
Time of follow-up	3.6 ± 0.9	3.4–3.8	4.0 ± 1.0	3.6–4.3	0.07
Total hip replacement	0 (0)	0–0	10 (37)	19–55	***p* < 0.001**
iHOT PASS < 60 points	0 (0)	0–0	17 (63)	45–81	***p* < 0.001**
iHOT score (points)	86 ± 12	84–89	44 ± 11*	38–50	***p* < 0.001**
Age (years)	32 ± 10	30–32	38 ± 9	34–42	**0.01**
Age over 40 (years)	17 (21)	12–30	14 (52)	33–71	**0.01**
Sex (%)	33 (41)	30–52	9 (33)	15–51	0.65
Tönnis grade of osteoarthritis > 0	23 (29)	19–39	16 (59)	40–78	**0.01**
Alpha angle on radial MRI (°)	70 ± 9	68–72	73 ± 11	69–78	0.15
Neck shaft angle (°)	129 ± 5	128–130	129 ± 4	127–130	0.25
Femoral torsion (°)	30 ± 10	28–32	25 ± 11	21–30	**0.046**
Lateral center edge angle (°)	28 ± 6	27–30	27 ± 5	25–29	0.56
Cross over sign	44 (55)	44–66	13 (48)	29–67	0.66
Posterior wall sign	44 (55)	44–66	14 (52)	33–71	> 0.99
Ischial spine sign	20 (25)	16–35	7 (26)	9–43	> 0.99

Bold letters indicate *p *< 0.05

^*^Mean values refer to the 17 hips that had not undergone total hip arthroplasty. *SD* standard deviation, *CI* confidence interval

### Association of MRI findings with failure of FAI surgery

Among the demographic parameters, patient age > 40 years was the only parameter associated with failure of FAI surgery with a HR of 2.4 (95% CI, 1.7 to 3.2; *p* = 0.02). On MRI, the presence of an acetabular cyst (*p* = 0.002) and femoral/acetabular osteophytes (excluding peripheral acetabular osteophyte; *p* = 0.15) were associated (all *p* < 0.05) with failure after FAI surgery in the univariate Cox regression (Table 3). Hazard ratios ranged from 2.5 (1.6 to 3.3, *p* = 0.034) for central acetabular osteophyte with a prevalence of 38% in the “good outcome” group and 70% in the “failure” group to hazard ratios of 5.0 (4.2 to 5.8, *p* < 0.001) for central femoral osteophyte with a prevalence of 18% in the ‘good outcome’ group and 70% in the ‘failure’ group, respectively. Soft tissue lesions associated with failure were labrum damage extending > 2 h (HR of 5.5, 4.7 to 6.4; *p* < 0.001) and acetabular cartilage damage > 2 h (HR of 5.1, 4.3 to 5.9;* p* < 0.001). The prevalence of these lesions was of 9%/5% in the “good outcome” group and of 37%/52% in the ‘failure’ group, respectively (Table 3).

**Table 3:** Predictive factors for failure* of arthroscopic hip surgery assessed with univariate Cox regression analysis

Parameter	Good outcome hips in % (95% CI)	Failure hips in % (95% CI)	Hazard ratio (95% CI)	*p* value
Number of hips	80 (75)	27 (25)	**-**	**-**
Age over 40 (years)	17 (21, 12–30)	14 (52, 33–71)	2.4 (1.7–3.2)	**0.02**
Tönnis grade of osteoarthritis > 0	23 (29, 19–39)	16 (59, 39–73)	2.1 (1.3–2.8)	0.06
Labrum damage extending > 2 h	7 (9, 3–15)	10 (37, 19–55)	5.5 (4.7–6.4)	** < 0.001**
Acetabular cartilage damage > 2 h	4 (5, 2–10)	14 (52, 33–71)	5.1 (4.3–5.9)	** < 0.001**
Femoral cartilage damage	11 (14, 6–21)	8 (30, 12–47)	2.3 (1.4–3.1)	0.06
Ligamentum teres damage	30 (38, 27–48)	13 (48, 29–67)	2.1 (1.2–2.9)	0.09
Peripheral acetabular osteophyte	59 (74, 64–84)	24 (89, 77–101)	2.5 (1.2–3.7)	0.15
Central acetabular osteophyte	30 (38, 27–49)	19 (70, 53–87)	2.5 (1.6–3.3)	**0.034**
Peripheral femoral osteophyte	32 (40, 29–51)	21 (78, 62–94)	3.1 (2.2–4.0)	**0.014**
Central femoral osteophyte	14 (18, 10–26)	19 (70, 62–78)	5.0 (4.2–5.8)	** < 0.001**
Acetabular cyst	20 (25, 16–35)	17 (63, 45–81)	3.4 (2.6–4.2)	**0.002**

Bold letters indicate *p* < 0.05

*CI* confidence interval, *failure was defined as an iHOT-12 of < 60 points or conversion to a total hip arthroplasty

When analyzing all parameters together in the multivariate Cox regression analysis, three negative predictors were independently associated with failure after FAI surgery: Acetabular cartilage damage > 2 h (HR of 3.2, 2.2 to 4.1; *p* = 0.01), central femoral osteophyte (HR of 3.1, 2.2 to 4.1; *p* = 0.02), and femoral cartilage damage with ligamentum teres damage (HR of 3.0, 1.9 to 4.1; *p* = 0.04) (Table 4).

**Table 4:** Independent predictive factors for failure* of arthroscopic hip surgery assessed

Parameter	Adjusted hazard ratio (95% CI)	*p* value
Acetabular cartilage damage > 2 h	3.2 (2.2–4.1)	**0.01**
Central femoral osteophyte	3.1 (2.2–4.1)	**0.02**
Femoral cartilage damage with ligamentum teres damage	3.0 (1.9–4.1)	**0.04**

Bold letters indicate *p* < 0.05

*CI* confidence interval. *failure was defined as an iHOT-12 of < 60 points or conversion to a total hip arthroplasty

**Fig. 3:**
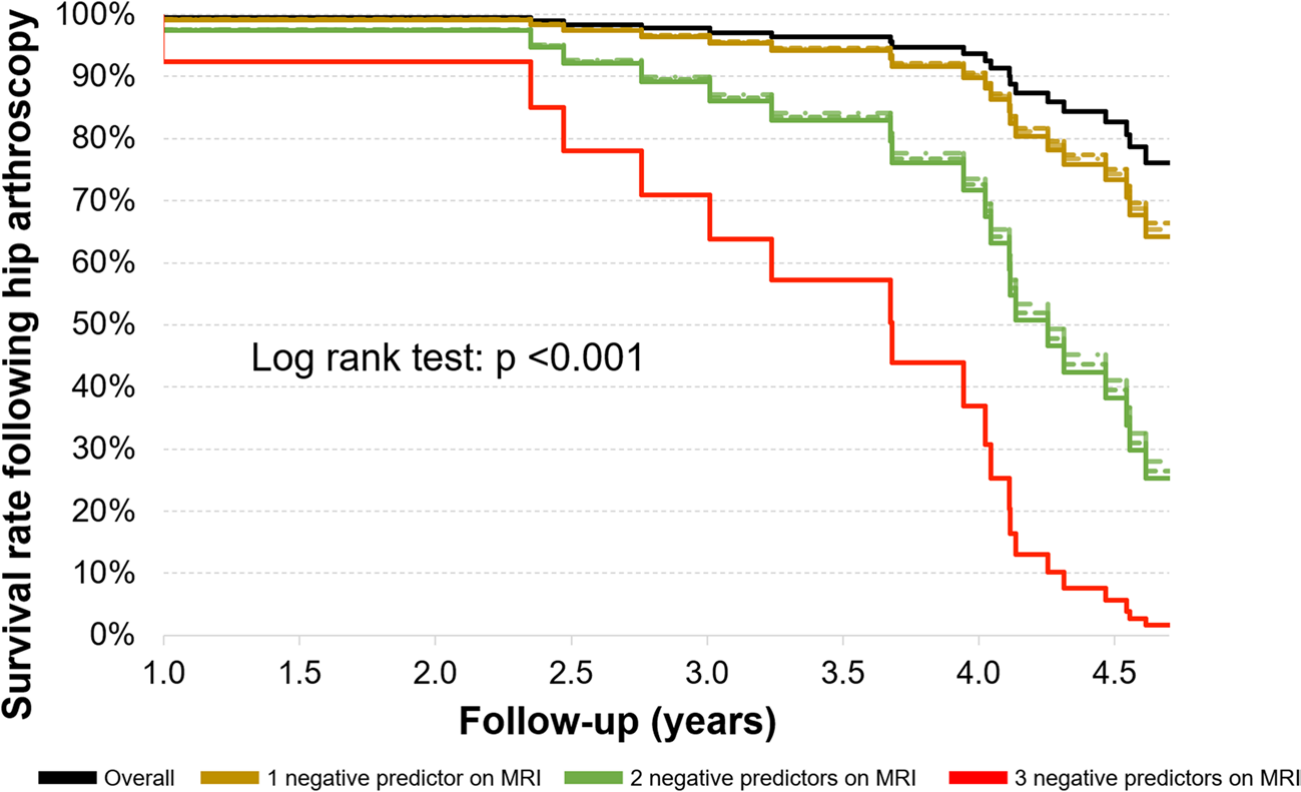
Survival rate decreases after hip arthroscopy based on the isolated or combined presence of negative predictors on traction MRI: acetabular cartilage damage > 2 h, central femoral osteophyte, femoral cartilage damage with associated ligamentum teres damage

**Fig. 4:**
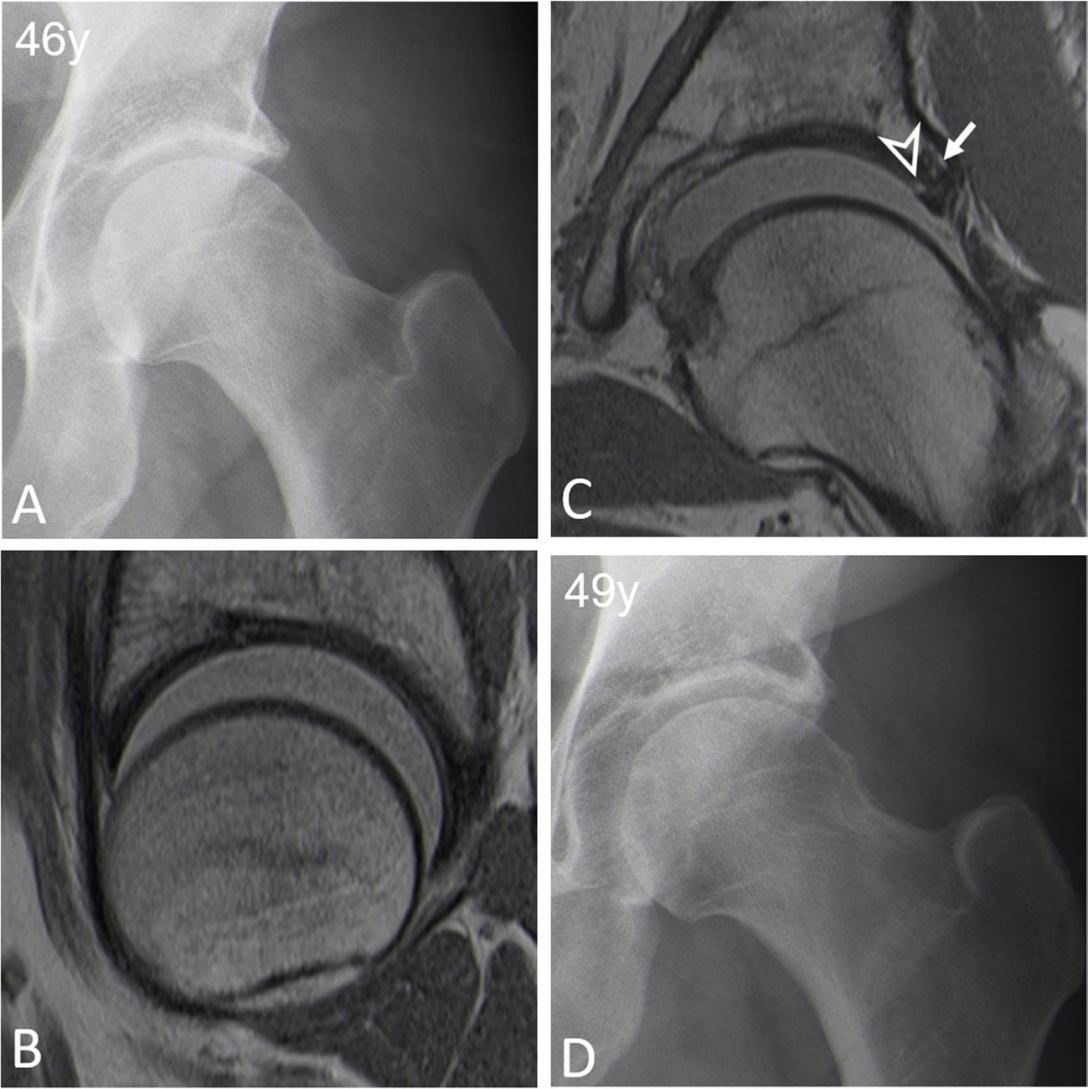
**A** AP pelvis view of a 46-year-old woman with mixed FAI. **B**, **C** Preoperative traction MR-arthrogram with proton density-weighted turbo spin echo images showing none of the MRI findings independently associated with failure of FAI surgery. **C** On the coronal image, only a focal acetabular cartilage lesion and (arrowhead) and peripheral acetabular osteophyte (arrow) are seen superiorly while the more anterior regions do not show any degenerative findings on the sagittal image (**B**). **D** AP pelvis view three years after arthroscopic femoral- and acetabular osteochondroplasty shows a preserved hip joint

**Fig. 5:**
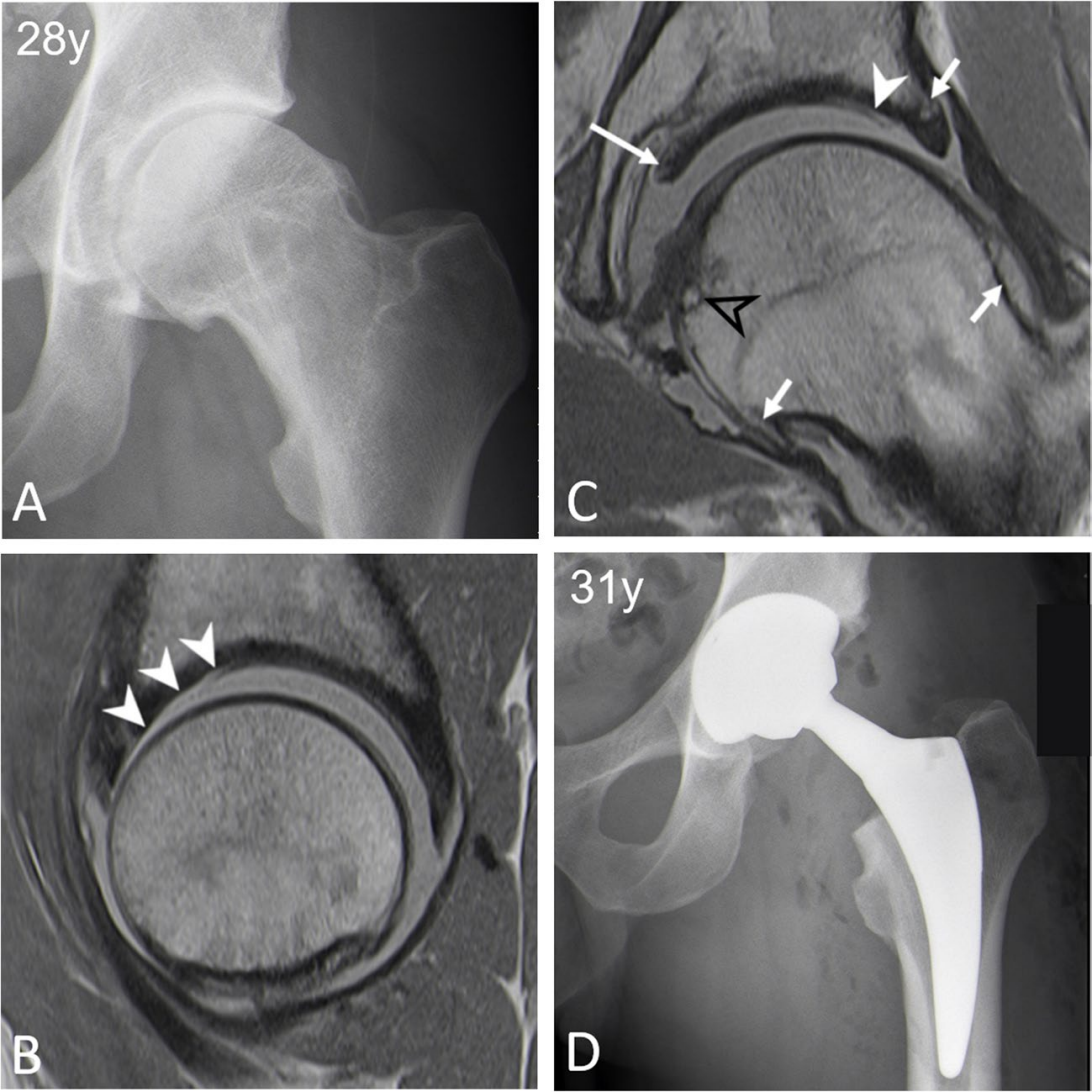
**A** 28-year-old man with cam FAI. **B** Preoperative traction MR-arthrogram with proton density-weighted turbo spin echo images showing two MRI findings independently associated with failure of FAI surgery. Sagittal- (**B**) and coronal (**C**) images show extensive cartilage defects involving the anterior- and superior quadrants (arrowheads). **C** The coronal image further demonstrates the central femoral osteophyte (black arrowhead), a central acetabular osteophyte (long arrow), as well as peripheral acetabular/ femoral osteophytes (short arrows). **D** Postoperative radiograph following total hip replacement 3 years after hip arthroscopy

**Fig. 6:**
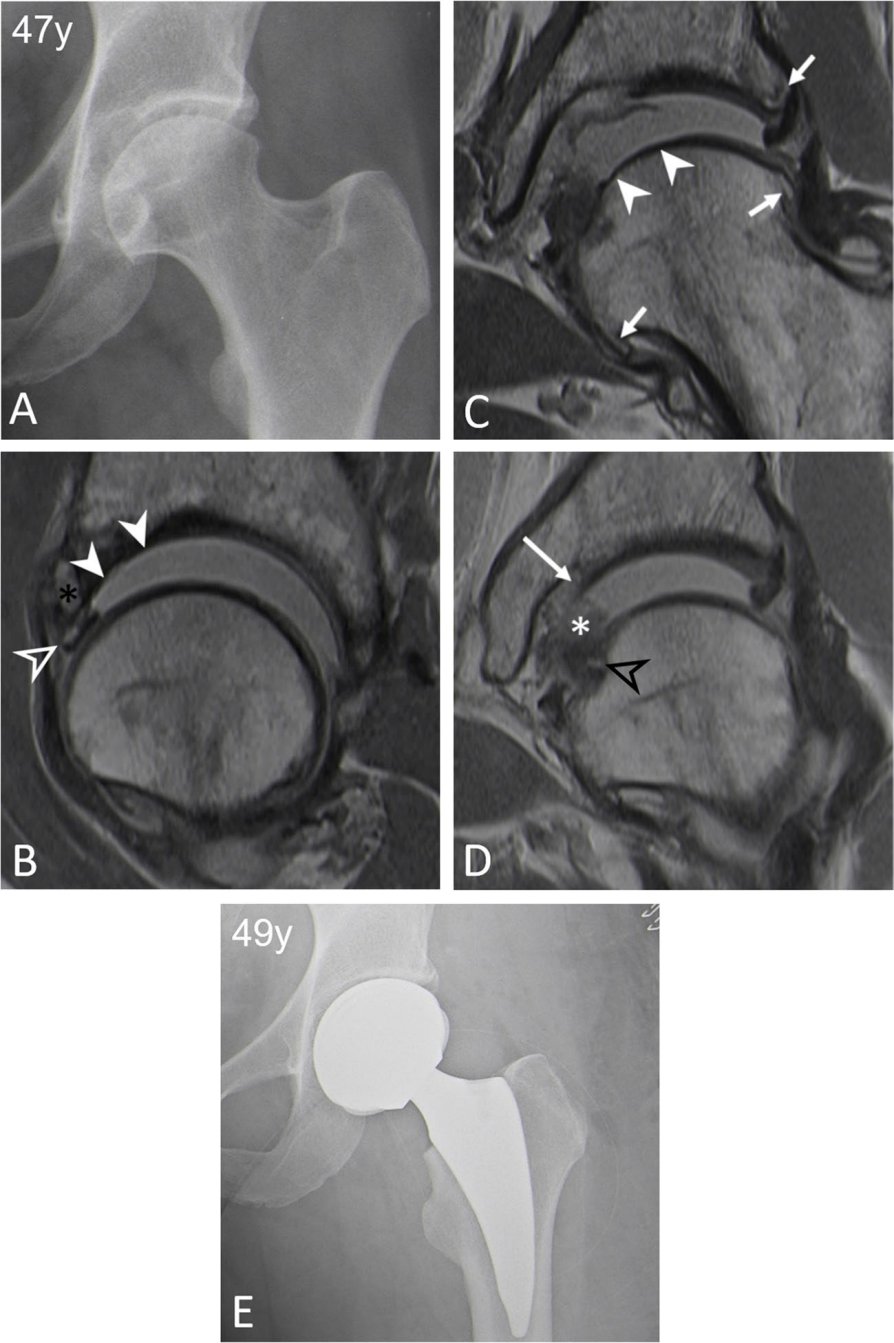
**A** 47-year-old woman with pincer FAI. **B**–**D** Preoperative traction MR-arthrogram with proton density-weighted turbo spin echo images showing three MRI findings independently associated with worse outcomes following FAI surgery. **B** The sagittal image shows an extensive cartilage defect > 2 h on the clockface (arrowheads), an associated anterior labrum lesion (empty arrowhead), and subchondral cyst formation at the acetabular rim (asterisk). **C**, **D** Consecutive coronal images demonstrate the central femoral osteophyte (black arrowhead) and a central femoral cartilage defect (arrowheads) with associated damage to the ligamentum teres (asterisk). In addition, a central acetabular osteophyte (long arrow), as well as peripheral acetabular/ femoral osteophytes (short arrows) are seen. **E** Postoperative radiograph following total hip replacement 2 years after the hip arthroscopy

Kaplan–Meier analysis revealed an overall survival rate of 75% at 4.6 years after arthroscopic FAI surgery that decreased with increasing prevalence of degenerative lesions (*p* < 0.001) (Figs. 3 and 4). When any one of the three independent negative MRI predictors was present, the survival decreased (66% for femoral cartilage damage with ligamentum teres damage, 65% for central femoral osteophyte, and 64% for acetabular damage > 2 h). When two out of three factors were present, survival further decreased to 25–28% (Fig. 5). When all three negative predictors were present on MRI, survival further decreased to 16% (Fig. 6).

### Interrater agreement

Interrater agreement was moderate for central femoral osteophyte (*κ* = 0.59; 95% CI, 0.43 to 0.74) to substantial, ranging from *κ* = 0.61 (0.45 to 0.76) for central acetabular osteophyte to *κ* = 0.78 (0.63 to 0.94) for acetabular cartilage > 2 h (Table 5).

**Table 5:** Inter-rater agreement for MRI parameters

Parameter	Cohen’s kappa	95% CI
Labrum damage extending > 2 h	0.69	0.52–0.87
Acetabular cartilage damage > 2 h	0.78	0.63–0.94
Femoral cartilage damage	0.72	0.54–0.90
Ligamentum teres damage	0.61	0.45–0.76
Peripheral acetabular osteophyte	0.67	0.49–0.85
Central acetabular osteophyte	0.75	0.63–0.88
Peripheral femoral osteophyte	0.63	0.48–0.77
Central femoral osteophyte	0.59	0.43–0.74
Acetabular cyst	0.77	0.64–0.90

*CI* confidence interval

## Discussion

Association between advanced age and advanced arthritis on radiographs are known risk factors for unfavorable outcomes of FAI surgery, yet the degenerative features on MRI that dictate osteoarthritis progression remain poorly understood [10]. Therefore, we aimed to illuminate the association between degenerative findings assessed with traction MRA and failure of arthroscopic FAI surgery.

Despite being considered typical findings of osteoarthritis, studies evaluating the association of osteophyte formation [10] and subchondral cysts [8, 9] with the outcome of FAI surgery are scarce. Hanke and colleagues [10] evaluated the association between osseous- and chondrolabral lesions on MRA performed at 1.5 T with failure of open FAI surgery at a mean 11-year follow-up. They demonstrated increased HR for posteroinferior femoral osteophytes (HR of 3.2, *p* = 0.01), central perifoveal osteophytes (HR of 3.9, *p* = 0.01), central “sabertooth” osteophytes of the acetabular fossa (HR of 4.0, *p* = 0.002), acetabular rim cysts (HR of 4.1, *p* = 0.01) and conversion to THA/ poor clinical outcome. Despite the shorter follow-up in our study (mean 3.7 years) and the different surgical approach (hip arthroscopy versus open surgical hip dislocation), we could confirm these findings. Central acetabular osteophytes (HR of 2.5; *p* = 0.034), peripheral- (HR of 3.1, *p* = 0.014) and central (HR of 5.0, *p* < 0.001) femoral osteophytes, and acetabular cysts (HR of 3.4, *p* = 0.002) were associated with failure of arthroscopic FAI correction.

Subchondral cysts supposedly result from bone resorption secondary to increased joint reacting forces resulting from advanced cartilage damage [22]. In our study, the frequency of acetabular cysts was higher (63% versus 25%, HR of 3.4; *p* = 0.002) in hips with subsequent failure. This is in line with a study on non-contrast MRI of the hip, which showed that the failure rate at 2 years was higher (33% versus 15%, *p* = 0.02) in hips with acetabular cysts compared to controls [9]. Similarly, Hartigan et al [8] reported conversion to THA in 33% and 16% of hips with femoral- and acetabular cysts on non-contrast MRI of the hip, respectively. While these studies found severe macroscopic cartilage damage intraoperatively in most patients with acetabular cysts, the authors did not evaluate further degenerative chondrolabral lesions on MRI [8, 9]. By contrast we found extensive labral damage > 2 h (HR of 5.5, *p* < 0.001) and acetabular cartilage damage > 2 h (HR of 5.1, *p* < 0.001) to be associated with failure after arthroscopic FAI correction. In addition, thanks to the application of traction, we could further differentiate between acetabular and femoral cartilage lesions. Compared to previous studies [8–10], we performed additional analysis, further including advanced age > 40 years and radiographic Tönnis grade > 0. Thereby, we were able to identify extensive acetabular cartilage damage > 2 h (HR of 3.2, *p* = 0.01), central femoral osteophytes (hazard ratio of 3.1, *p* = 0.02), as well as femoral cartilage- associated with ligamentum teres damage (hazard ratio 3.0, *p* = 0.04) as independent risk factors. Interestingly, two of these three negative predictors identified reflect a pattern of central hip joint degeneration previously not described on MRI. This finding supports intraoperative observations suggesting that central femoroacetabular osteophytes lead to degeneration of the ligamentum teres and secondary femoral cartilage wear. This pattern of degeneration differs from the initial stages of FAI, in which the damage is confined to the chondrolabral transition zone [23].

Recently, the added value of preoperative routine MRI of the hip was questioned in patients younger than 40 years due to the lack of data supporting its prognostic value [24]. However, since the short-term failure rates following arthroscopic FAI surgery may be as high as 18%, there is a need for improved patient selection aside from demographic risk factors such as advanced age [5]. We could demonstrate that extensive acetabular cartilage damage > 2 h, central femoral osteophytes as well as femoral cartilage- associated with ligamentum teres damage was associated with failure independently from age > 40/ < 40 years. In addition, the calculated probability of prothesis-free survival/ good clinical outcome progressively deteriorated when more than one of these findings was present (Figs. 3 and 6). This underlines the potential role of degenerative MRI findings to improve risk stratification and management of patient expectations in typically young patients with FAI (Fig. 5). Furthermore, the absence of these degenerative findings may serve as a helpful adjunct for patient selection in patients aged > 40 years who are already at a higher risk for failure of FAI surgery (Fig. 4).

The limitations of our study are related to its retrospective nature. First, a significant number of patients were lost to follow-up. This can potentially introduce a selection bias as either patients with better or worse outcomes than the ones included have been missed. Second, during the study period, the institutional protocol for hip MRA was changed to injection of 0.9% saline solution instead of diluted gadolinium contrast. The proportion of hips with either protocol was not different between outcome groups (48% in the failure group, 45% in the good outcome group; *p* = 0.70). Furthermore, a previous study showed comparable results regarding image quality and diagnostic accuracy for saline- and gadolinium-based MRA [25]. Thus, this should not jeopardize our findings to a relevant degree. Third, a comparison between non-contrast MRI, conventional MRA, and traction MRA of the hip in the same patient was not possible due to time constraints. However, our findings demonstrate the prognostic potential of femoral and central osteophytes as well as acetabular cysts in predicting outcomes. Since detection of these lesions is not affected by injection of contrast media or application of traction, they may be applied to non-contrast MRI and conventional MR arthrography of the hip. Despite that, prospective studies with longer follow-up are necessary to confirm our findings using different imaging techniques.

To conclude, we could identify MRI parameters that can potentially aid surgeons when contemplating which patients can still benefit from arthroscopic FAI surgery based on the severity of already present joint degeneration. Among these parameters, extensive acetabular cartilage damage > 2 h, central femoral osteophytes, and femoral cartilage damage associated with ligamentum teres damage were associated with failure, independent of advanced age and radiographic arthritis.

## Abbreviations

CI: Confidence interval
FAI: Femoroacetabular impingement
HR: Hazard ratio
iHOT-12: International Hip Outcome Tool
LCE: Lateral center-edge
MRA: Magnetic resonance arthrography
MRI: Magnetic resonance imaging
THA: Total hip arthroplasty

## Declarations

### Guarantor

The scientific guarantor of this publication is Florian Schmaranzer, MD PhD.

### Statistics and biometry

One of the authors (Jospeh M. Schwab, MD) has significant statistical expertise.

### Study subjects or cohorts overlap

No study subjects or cohorts have been previously reported.

### Methodology

• retrospective

• observational

• performed at one institution
